# Metformin Reduces Thyroid Cancer Risk in Taiwanese Patients with Type 2 Diabetes

**DOI:** 10.1371/journal.pone.0109852

**Published:** 2014-10-10

**Authors:** Chin-Hsiao Tseng

**Affiliations:** 1 Department of Internal Medicine, National Taiwan University College of Medicine, Taipei, Taiwan; 2 Division of Endocrinology and Metabolism, Department of Internal Medicine, National Taiwan University Hospital, Taipei, Taiwan; 3 Division of Environmental Health and Occupational Medicine of the National Health Research Institutes, Taipei, Taiwan; Kagoshima University Graduate School of Medical and Dental Sciences, Japan

## Abstract

**Background:**

Whether metformin may affect thyroid cancer risk has not been studied. This study investigated the association between metformin use and thyroid cancer risk in Taiwanese patients with type 2 diabetes mellitus.

**Methods:**

The reimbursement databases of all diabetic patients from 1996 to 2009 were retrieved from the National Health Insurance. An entry date was set at 1 January 2006 and 1,414,723 patients with type 2 diabetes were followed for thyroid cancer incidence until the end of 2009. Incidences for ever-users, never-users and subgroups of metformin exposure using tertile cutoffs for cumulative duration of therapy and cumulative dose were calculated and adjusted hazard ratios were estimated by Cox regression. Additional sensitivity analyses were conducted.

**Results:**

There were 795,321 ever-users and 619,402 never-users, with respective numbers of incident thyroid cancer of 683 (0.09%) and 1,614 (0.26%), and respective incidence of 24.09 and 87.33 per 100,000 person-years. The overall fully adjusted hazard ratio (95% confidence interval) was 0.683 (0.598–0.780), and all categories of the dose-response parameters showed significantly lower risk with *P*-trends <0.0001. The protective effect of metformin on thyroid cancer incidence was also supported by sensitivity analyses, disregarding age (<50 or ≥50 years) and sex; and was not affected by excluding users of insulin, sulfonylurea, and insulin and/or sulfonylurea respectively, by previous diagnosis of other cancers or by potential detection examinations that might lead to differential diagnosis of thyroid cancer.

**Conclusions:**

This study provides evidence for the first time that metformin use in patients with type 2 diabetes may reduce the risk of thyroid cancer.

## Introduction

Patients with type 2 diabetes have a higher risk of cancer involving the breast, endometrium, stomach, colorectum, liver, pancreas, urinary bladder, and non-Hodgkin's lymphoma [1]–[8]. The mechanisms may be related to insulin resistance, hyperinsulinemia, proinflammatory status and increased oxidative stress [1], [2], [9]. With regards to thyroid cancer, there has been an increasing trend of its incidence over the past decades worldwide; and this has been ascribed to the increasing prevalence of insulin resistance [10].

Although insulin resistance may lead to an increased risk of thyroid cancer in patients with type 2 diabetes, our recent population-based study did not support a higher risk of thyroid cancer in these patients [11]. Recent studies suggested that antidiabetic drugs may play some role in the development of cancers. For example, metformin may reduce [12]–[16], while insulin and sulfonylureas may increase [1], [17]–[19] the risk of various types of cancer. On the other hand, the use of pioglitazone [20]–[22], but not rosiglitazone [21]–[23], may increase the risk of bladder cancer. Therefore, a lack of association between diabetes and thyroid cancer as observed in our previous study [11] can be a composite outcome of the interaction between the mechanisms that lead to a higher risk (e.g., insulin resistance) and the effects of the use of metformin that lead to a lower risk.

Whether metformin use can reduce the risk of thyroid cancer in patients with type 2 diabetes remains to be confirmed. Some recent *in vitro* studies suggested that metformin may inhibit the growth of thyroid cancer cells through inhibition of the mammalian target of rapamycin (mTOR) pathway by activating the AMP-activated protein kinase (AMPK) [24] or by diminishing the growth stimulation by insulin [25]. In humans, an earlier observational report of 4 cases showed that metformin might suppress thyroid stimulating hormone (TSH) without causing clinical hyperthyroidism [26], but this can not be confirmed in a later large sample-sized retrospective study comparing 250 patients on metformin therapy to 578 diabetic patients without metformin treatment [27]. A recent small clinical study conducted in 66 women with insulin resistance and thyroid nodular hyperplasia for 6 months suggested that metformin alone or better in combination with levothyroxine may reduce the size of the nodule [28].

All of the above *in vitro* and preliminary human studies suggested a potential usefulness of metformin on the prevention of thyroid cancer. However, large human observational studies are still lacking [16], [29]. Therefore, the purpose of the present study was to evaluate whether metformin use in Taiwanese patients with type 2 diabetes could affect the risk of thyroid cancer by using the National Health Insurance (NHI) database obtained from the whole nation.

## Materials and Methods

A retrospective cohort analysis using the NHI databases including all patients with a diagnosis of diabetes during the period from 1996 (the earliest database available) to 2009 in Taiwan was conducted. The study using the NHI database was approved by an ethic review board of the National Health Research Institutes with registered approval number 99274. Written informed consent from the participants was not required according to local regulations because the identification information of the individuals was scrambled and de-identified prior to analysis for the protection of privacy.

Since March 1995 a compulsory and universal system of health insurance (the so-called NHI) was implemented in Taiwan. All contracted medical institutes must submit computerized and standard claim documents for reimbursement. More than 99% of citizens are enrolled in the NHI, and >98% of the hospitals nationwide are under contract with the NHI. The average number of annual physician visits in Taiwan is one of the highest around the world, at approximately 15 visits per year per capita in 2009.

The databases contain detailed records on every visit for each patient, including outpatient visits, emergency department visits and hospital admission; and include principal and secondary diagnostic codes, prescription orders, and claimed expenses. Diabetes was coded 250.00–250.93 and thyroid cancer 193, based on the *International Classification of Diseases, Ninth Revision, Clinical Modification* (ICD-9-CM).

We first retrieved the databases of all patients who had been diagnosed as having diabetes and/or under treatment with either oral anti-diabetic agents or insulin during the period of 1996–2009 from the whole nation (*n* = 3,756,511). The selected entry date was 1 January 2006. After excluding patients who had a diagnosis of diabetes after the year 2006 (*n* = 773,801), patients who held a Severe Morbidity Card as having type 1 diabetes (*n* = 7182, in Taiwan, patients with type 1 diabetes were issued a so-called “Severe Morbidity Card” after certified diagnosis and they were waived for much of the co-payments), patients having a diagnosis of thyroid cancer before 2006 (*n* = 8,252), those who died (*n* = 169,509) or withdrew from the NHI (*n* = 23,784) before entry date, duplicated identification number (*n* = 216), unclear information on date of birth or sex (*n* = 14,284), and diabetic patients without any reimbursement record after the entry date (*n* = 1,556,791), a total of 1,414,723 patients with a diagnosis of type 2 diabetes and/or under therapy with oral anti-diabetic agents or insulin were recruited.

Those who had ever been prescribed metformin before entry date were defined as ever-users; and never-users were defined as those who had never been prescribed metformin before entry date. To evaluate whether a dose-response relationship could be seen between metformin and thyroid cancer, the tertile cutoffs for the following parameters were applied: 1) cumulative duration of therapy in months; and 2) cumulative dose in mg.

Baseline characteristics of ever- and never-users of metformin at entry were compared by Chi square test. Age and all comorbidities and covariates were determined as a status/diagnosis before the entry date. The ICD-9-CM codes for the comorbidities were [11]–[14], [30]: nephropathy 580–589, hypertension 401–405, chronic obstructive pulmonary disease (a surrogate for smoking) 490–496, stroke 430–438, ischemic heart disease 410–414, peripheral arterial disease 250.7, 785.4, 443.81 and 440–448, eye disease 250.5, 362.0, 369, 366.41 and 365.44, obesity 278, dyslipidemia 272.0–272.4, benign thyroid disease 240–246, and cancer other than thyroid cancer 140–208 (excluding 193). Medications included sulfonylurea, insulin, acarbose, pioglitazone, rosiglitazone, statin, fibrate, angiotensin-converting enzyme inhibitor and/or angiotensin receptor blocker, calcium channel blocker, aspirin, ticlopidine, clopidogrel, dipyridamole and non-steroidal anti-inflammatory drugs (excluding aspirin).

The incidence density of thyroid cancer was calculated for ever-users and never-users and for different subgroups of exposure. The numerator for the incidence was the number of patients with incident thyroid cancer during the 4-year follow-up from January 1, 2006 to December 31, 2009, and the denominator was the person-years of follow-up. For ever-users, the follow-up duration was either censored at the date of thyroid cancer diagnosis or at the date of the last record of the available reimbursement databases in individuals without incident thyroid cancer. In the lack of information on the vital status or migration of the patients, the last reimbursement record may serve as a surrogate because patients who die or migrate out of the country should be withdrawn from the NHI in Taiwan. For never-users, the follow-up was censored at the date of metformin initiation or thyroid cancer diagnosis or the last reimbursement record, depending on whichever occurring first. This ensured no exposure to metformin throughout the whole follow-up period until censor in the referent group of never-users.

Cox proportional hazards regression was performed to estimate the hazard ratios for thyroid cancer for ever-users versus never-users, and for the various subgroups of dose-response parameters. The following fully adjusted models were created with consideration of all covariates mentioned above: 1) Models I conducted in the whole original cohort; 2) Models II conducted after excluding users of both metformin and sulfonylurea, in order to avoid the potential bias resulted from multi-collinearity because a very high proportion of metformin users were also users of sulfonylurea; and 3) Models III conducted after excluding users of antidiabetic drugs other than metformin, in order to create a “cleaner” comparison for metformin only versus diabetic patients without use of antidiabetic drugs (i.e., on diet control only).

Additional sensitivity analyses were conducted by creating the following Cox regression models to estimate the fully adjusted hazard ratios for metformin users versus non-users: 1) In subgroups of men, women, age <50 years and age ≥50 years, respectively; 2) Excluding patients with a diagnosis of cancer other than thyroid cancer; 3) Excluding users of insulin, sulfonylurea, and insulin and/or sulfonylurea, respectively; 4) Additional adjustment for potential detection examinations including thyroid sonography, thyroid aspiration and thyroid function test (described previously [30]) that might have led to differential detection of thyroid cancer between ever-users and never-users of metformin.

Analyses were conducted using SAS statistical software, version 9.3 (SAS Institute, Cary, NC). *P*<0.05 was considered statistically significant.

## Results

Among the patients 317,508 (22.4%) were on diet control without any pharmacological intervention, 907,599 (64.2%) were using oral antidiabetic drugs only, 179,847 (12.7%) were using oral antidiabetic drugs plus insulin, and 9,769 (0.7%) were using insulin only.

Table 1 compares the baseline characteristics between ever- and never-users of metformin. All variables differed significantly between the two groups. Ever-users are characterized by older age distribution, higher proportion with a diabetes duration >5 years, higher proportions of all comorbidities except with a lower proportion of a diagnosis with other cancer, and higher proportions of using other medications.

**Table 1:** Baseline characteristics between never-users and ever-users of metformin.

Variables	Metformin				*P* value
	Never users		Ever users		
	*n*	%	*n*	%	
*n* = 1414723	619402		795321		
Age (years)					
<0	43229	6.98	30708	3.86	<0.0001
40–49	90027	14.53	100815	12.68	
50–59	155055	25.03	210883	26.52	
60–69	143566	23.18	214320	26.95	
≥70	187525	30.28	238595	30.00	
Sex (men)	291424	47.05	397316	49.96	<0.0001
Diabetes duration (years)					
<1	76021	12.27	39395	4.95	<0.0001
1–3	122049	19.70	97004	12.20	
3–5	109316	17.65	108390	13.63	
>5	312016	50.37	550532	69.22	
Hypertension	193690	31.27	536498	67.46	<0.0001
Chronic obstructive pulmonary disease	43116	6.96	149644	18.82	<0.0001
Stroke	37674	6.08	151546	19.05	<0.0001
Nephropathy	45351	7.32	124597	15.67	<0.0001
Ischemic heart disease	57508	9.28	238766	30.02	<0.0001
Peripheral arterial disease	21408	3.46	135384	17.02	<0.0001
Eye disease	10859	1.75	106035	13.33	<0.0001
Obesity	4015	0.65	15647	1.97	<0.0001
Dyslipidemia	196607	31.74	466909	58.71	<0.0001
Benign thyroid disease	21855	3.53	43401	5.46	<0.0001
Other cancer	106265	17.16	93978	11.82	<0.0001
Statin	63535	10.26	309483	38.91	<0.0001
Fibrate	40065	6.47	221696	27.88	<0.0001
Angiotensin converting enzyme inhibitor/angiotensin receptor blocker	101931	16.46	451197	56.73	<0.0001
Calcium channel blocker	83733	13.52	329741	41.46	<0.0001
Sulfonylurea	149415	24.12	722015	90.78	<0.0001
Insulin	17198	2.78	172418	21.68	<0.0001
Acarbose	14592	2.36	170206	21.40	<0.0001
Pioglitazone	2716	0.44	55662	7.00	<0.0001
Rosiglitazone	8381	1.35	165822	20.85	<0.0001
Aspirin	67805	10.95	320342	40.28	<0.0001
Ticlopidine	4643	0.75	29727	3.74	<0.0001
Clopidogrel	5491	0.89	32008	4.02	<0.0001
Dipyridamole	40354	6.51	210556	26.47	<0.0001
Non-steroidal anti-inflammatory drugs (excluding aspirin)	229967	37.13	596320	74.98	<0.0001

Table 2 shows the incidences of thyroid cancer between ever-users and never-users of metformin, and among the different categories of the dose-response parameters for metformin exposure. During follow-up, a total of 2,297 (0.16%) patients developed thyroid cancer. Among them, 1,614 were never-users and 683 were ever-users of metformin at entry. The incidence rate in never- and ever-users of metformin was 87.33 and 24.09 per 100,000 person-years, respectively.

**Table 2:** Incidence of thyroid cancer by metformin exposure at entry.

Metformin use	Case number	Incident thyroid cancer	%	Person-years	Incidence rate
					(per 100,000 person-years)
Never users	619402	1614	0.26	1848266.92	87.33
Ever users	795321	683	0.09	2835264.08	24.09
*P* value			0.0222		
**Cumulative duration of therapy (months)**					
Never users	619402	1614	0.26	1848266.92	87.33
<9.13	262339	257	0.10	905836.25	28.37
9.13–37.00	262486	211	0.08	945139.58	22.32
>37.00	270496	215	0.08	984288.25	21.84
*P* value			0.0067		
**Cumulative dose (mg)**					
Never users	619402	1614	0.26	1848266.92	87.33
<264,000	262425	258	0.10	908265.75	28.41
264,000–1,263,000	262430	217	0.08	943853.92	22.99
>1,263,000	270466	208	0.08	983144.42	21.16
*P* value			0.0043		

Table 3 shows the fully adjusted hazard ratios with regards to different categories of metformin exposure in Models I, II and III. In the models evaluating the overall hazard ratios for ever-users versus never-users, all of the hazard ratios showed a significantly lower risk associated with metformin use. In the models evaluating the dose-response exposure to metfomrin, a lower risk could be seen in all tertiles of the dose-response parameters, with significant *P*-values for the trend. The dose-response effect in Models I was not as remarkable as in Models II, suggesting potentially biased estimates resulting from the high correlation between use of metformin and sulfonylurea in Models I.

**Table 3:** Metformin exposure at entry and hazard ratios for thyroid cancer.

Metformin use	Models I			Models II			Models III		
	HR	95% CI	*P*	HR	95% CI	*P*	HR	95% CI	*P*
Ever-users	0.683	(0.598–0.780)	<0.0001	0.421	(0.334–0.530)	<0.0001	0.432	(0.336–0.555)	<0.0001
**Cumulative duration of therapy (months)**									
<9.1	0.699	(0.601–0.813)	<0.0001	0.439	(0.336–0.574)	<0.0001	0.461	(0.347–0.611)	<0.0001
9.1–36.9	0.655	(0.547–0.785)	<0.0001	0.396	(0.249–0.630)	<0.0001	0.426	(0.253–0.717)	0.0013
>36.9	0.677	(0.554–0.826)	0.0001	0.339	(0.150–0.765)	0.0092	0.159	(0.039–0.642)	0.0098
*P*-trend			<0.0001			<0.0001			<0.0001
**Cumulative dose (mg)**									
<263,000	0.691	(0.594–0.803)	<0.0001	0.433	(0.333–0.564)	<0.0001	0.439	(0.331–0.583)	<0.0001
263,000–1,260,000	0.675	(0.564–0.808)	<0.0001	0.408	(0.257–0.650)	0.0002	0.475	(0.287–0.788)	0.0039
>1,260,000	0.667	(0.543–0.819)	0.0001	0.314	(0.117–0.848)	0.0222	0.123	(0.017–0.880)	0.0369
*P*-trend			<0.0001			<0.0001			<0.0001

Referent group: never-users of metformin; HR: hazard ratio, CI: confidence interval.

Models I: Fully adjusted models conducted in the whole cohort.

Models II: Fully adjusted models conducted after excluding users of both metformin and sulfonylurea.

Models III: Fully adjusted models conducted after excluding users of antidiabetic drugs other than metformin.

Table 4 shows the fully adjusted hazard ratios for thyroid cancer in the additional sensitivity analyses. The results suggested that the protective effect of metformin on thyroid cancer was not restricted to a certain group of age or sex; and was not affected by excluding users of insulin, sulfonylurea, and insulin/and or sulfonylurea, respectively, by previous diagnosis of other cancers or by potential detection examinations.

**Table 4:** Additional sensitivity analyses for metformin and thyroid cancer.

Model	Fully adjusted HR	95% CI	*P*-value
Men	0.770	(0.602–0.985)	0.0379
Women	0.651	(0.555–0.762)	<0.0001
Age <50 years	0.576	(0.437–0.759)	<0.0001
Age ≥50 years	0.732	(0.628–0.853)	<0.0001
Excluding patients with a diagnosis of cancers other than thyroid cancer	0.613	(0.538–0.699)	<0.0001
Excluding users of insulin	0.682	(0.594–0.782)	<0.0001
Excluding users of sulfonylurea	0.434	(0.344–0.548)	<0.0001
Excluding users of insulin and/or sulfonylurea	0.436	(0.344–0.552)	<0.0001
Additional adjustment for potential detection examination	0.667	(0.584–0.761)	<0.0001

Referent group: never-users of metformin.

HR: hazard ratio, CI: confidence interval.

## Discussion

Observational studies suggested that metformin may be associated with a reduced risk of some cancer involving the bladder, breast, liver, and colorectum [14]–[16]. However, whether metformin use can be associated with a reduced risk of thyroid cancer has not been investigated previously [16], [29]. This is the first observational study that clearly demonstrated a protective effect of metformin on thyroid cancer risk in patients with type 2 diabetes (Tables 2, 3 and 4).

Several randomized clinical trials are being conducted to evaluate the potential usefulness of metformin as an adjuvant to other chemotherapeutic agents on some solid cancers like the breast, endometrial, prostate, and lung cancer [31]. However, clinical trials focusing specifically on the effect of metformin on thyroid cancer are still lacking. This study provided an important rationale for future in-depth investigation on metformin for the prevention and treatment of thyroid cancer.

A large number (*n* = 1,556,791) of the patients with a diagnosis of diabetes did not have reimbursement data after study entry and had been excluded from the analyses. This could be a main source of bias in the study. It is assumable that this population of excluded diabetic patients might have mainly composed of patients being on diet control without any pharmacological intervention. The consistent finding in the comparison of metformin-treated patients to patients on diet control alone conducted in Models III of Table 3 would obviate the concern of this potential bias.

The present study suggested that a significantly lower risk of thyroid cancer could be observed with a cumulative duration of 9 months or a cumulative dose of 263,000 mg (Table 3). This time window for a protective effect of metformin may be supported by a small clinical trial showing that metformin use for 6 months may significantly reduce the size of thyroid nodules [28], and by another study showing that the adjusted hazard ratio for prostate cancer-specific mortality was 0.76 (95% confidence interval: 0.64–0.89) for each additional 6 months of metformin use [32].

If insulin resistance is the underlying cause of thyroid cancer as advocated by Gursoy [10] and metformin treatment in patients with type 2 diabetes would reduce the incidence of thyroid cancer (Tables 2, 3 and 4), it is expected that the incidence of thyroid cancer should be increased gradually from obesity, impaired glucose tolerance through the early stage of type 2 diabetes when they were put on diet control without pharmacological intervention, but the risk would decrease when metformin was initiated. However, such a temporal change in thyroid cancer incidence becomes more complicated when the disease progresses to the development of diabetes complications, when other clinical comorbidities set in, or when multiple drugs are used. Because most patients with type 2 diabetes require multiple antidiabetic drugs for controlling their blood glucose [33], the joint effects and interactions among the various antidiabetic drugs are interesting and important. However, these are out of the scope of the present study and await future investigations.

The anti-cancer effects of metformin may probably involve the activation of the AMPK, inhibition of the mTOR pathway, and inhibition of insulin-like growth factors [34]. All of these have been demonstrated in *in vitro* studies conducted in thyroid cancer cell lines [24], [25], in women with insulin resistance and benign thyroid nodules [28] and in patients with type 2 diabetes [35]. The suppression on TSH by metformin observed in humans [26], [36] may also contribute to the lower risk of thyroid cancer in metformin users, although this beneficial effect can not be confirmed by another recent study [27].

Leptin, a hormone produced by adipocytes and regulates energy balance in the brain, signals the expression of thryotropin-releasing hormone, leading to increased secretion of TSH [37]. Therefore, one of the possible mechanisms explaining the inhibitory effect of metformin on TSH secretion may be through its leptin inhibition [37]. Recent basic researches also suggested that metformin may sensitize thyroid cancer cells to oxidative stress [38], inhibit thyroid cancer stem cells [25], [39], cause cell cycle arrest at G0/G1 phase [25], induce apoptosis [25], inhibit colony formation [25], inhibit stimulation of mitogen-activated protein kinase by insulin [25], and inhibit growth of doxorubicin-resistant thyroid carcinoma cells [25]. The growth inhibitory effect of metformin is not only demonstrated in thyroid carcinoma cells, a recent study also demonstrates such a growth inhibitory effect on medullary thyroid cancer cells [24]. Metformin may have an advantage of inducing growth arrest specifically in cancer stem cells through the inhibition of Akt, which is not affected in differentiated cells [40], [41].

There are several strengths in the present study. First, we made a special request to include all patients diagnosed as having diabetes from the NHI database covering the whole period since its availability in 1996. Such an approach avoided the possibility of selection bias and insufficient cases of thyroid cancer for subgroup analyses. In the absence of a special request, the usually provided NHI database is a 1 million random sample of the general population, and the number of diabetic patients derived from such a 1 million sample is surely too small for the analyses of the association between the use of anti-diabetic drugs and some cancers. Second, the consistency of a lower risk of thyroid cancer in metformin users in the sensitivity analyses strengthened the preventive role of metformin on thyroid cancer. Third, the database included outpatients and inpatients, and we included diagnoses from both sources. The use of medical records also reduced the bias related to self-reporting.

The limitations of the study may include a lack of the histological types of thyroid cancer for analysis. However, because thyroid papillary cancer represents 80.5% and 87.1% of thyroid cancer in men and women, respectively, in Taiwan [42], the findings should better be applied to thyroid papillary cancer. Second, although some cases of thyroid cancer may have been misclassified, such an occurrence was probably low because labeled diagnoses should be printed on all prescriptions handed out to patients in Taiwan. Mislabeling of a cancer diagnosis would not be acceptable to the patients when they saw the diagnosis. Third, ionizing radiation has been implicated as a risk factor for thyroid cancer [43]. A confounding effect due to the lack of such data collection could not be excluded. Fourth, we did not have biochemical data for evaluating their impacts.

In summary, this study supports a protective effect of metformin on the development of thyroid cancer in Taiwanese patients with type 2 diabetes. The uses of metformin for reversing the increasing trend of thyroid cancer incidence in the general population and for the treatment of thyroid cancer are worthy of further investigation.

## References

[1] Arcidiacono B, Iiritano S, Nocera A, Possidente K, Nevolo MT, et al (2012) Insulin resistance and cancer risk: an overview of the pathogenetic mechanisms. Exp Diabetes Res 2012: 789174. 22701472 10.1155/2012/789174 PMC3372318

[2] Nicolucci A (2010) Epidemiological aspects of neoplasms in diabetes. Acta Diabetol 47: 87–95. 20376506 10.1007/s00592-010-0187-3

[3] Tseng CH (2011) Diabetes conveys a higher risk of gastric cancer mortality despite an age-standardised decreasing trend in the general population in Taiwan. Gut 60: 774–779. 21193459 10.1136/gut.2010.226522

[4] Tseng CH, Chong CK, Tseng CP, Chan TT (2009) Age-related risk of mortality from bladder cancer in diabetic patients: A 12-year follow-up of a national cohort in Taiwan. Ann Med 41: 371–379. 19191082 10.1080/07853890902729778

[5] Tseng CH (2011) Diabetes and risk of bladder cancer: A study using the National Health Insurance database in Taiwan. Diabetologia 54: 2009–2015. 21544514 10.1007/s00125-011-2171-z

[6] Tseng CH (2012) Diabetes and non-Hodgkin's lymphoma: Analyses of prevalence and annual incidence in 2005 using the National Health Insurance database in Taiwan. Ann Oncol 23: 153–158. 21765043 10.1093/annonc/mdr334

[7] Tseng CH (2012) Diabetes, insulin use, and non-Hodgkin lymphoma mortality in Taiwan. Metabolism 61: 1003–1009. 22237115 10.1016/j.metabol.2011.11.015

[8] Tseng CH (2013) New-onset diabetes with a history of dyslipidemia predicts pancreatic cancer. Pancreas 42: 42–48. 22750971 10.1097/MPA.0b013e3182571ba9

[9] Dong X, Li Y, Tang H, Chang P, Hess KR, et al (2012) Insulin-like growth factor axis gene polymorphisms modify risk of pancreatic cancer. Cancer Epidemiol 36: 206–211. 21852217 10.1016/j.canep.2011.05.013

[10] Gursoy A (2010) Rising thyroid cancer incidence in the world might be related to insulin resistance. Med Hypotheses 74: 35–36. 19720470 10.1016/j.mehy.2009.08.021

[11] Tseng CH (2012) Thyroid cancer risk is not increased in diabetic patients. PLoS One 7: e53096. 23300866 10.1371/journal.pone.0053096 PMC3531389

[12] Tseng CH (2012) Diabetes, metformin use, and colon cancer: A population-based cohort study in Taiwan. Eur J Endocrinol 167: 409–416. 22778198 10.1530/EJE-12-0369

[13] Tseng CH (2013) Benign prostatic hyperplasia is a significant risk factor for bladder cancer in diabetic patients: a population-based cohort study using the National Health Insurance in Taiwan. BMC Cancer 13: 7. 23286275 10.1186/1471-2407-13-7 PMC3541059

[14] Tseng CH (2014) Metformin may reduce bladder cancer risk in Taiwanese patients with type 2 diabetes. Acta Diabetol 51: 295–303. 24509842 10.1007/s00592-014-0562-6

[15] Tseng CH (2014) Metformin may reduce breast cancer risk in Taiwanese women with type 2 diabetes. Breast Cancer Res Treat 145: 785–790. 24816805 10.1007/s10549-014-2985-8

[16] Franciosi M, Lucisano G, Lapice E, Strippoli GF, Pellegrini F, et al (2013) Metformin therapy and risk of cancer in patients with type 2 diabetes: systematic review. PLoS One 8: e71583. 23936520 10.1371/journal.pone.0071583 PMC3732236

[17] Tseng CH (2013) Diabetes, insulin use, smoking, and pancreatic cancer mortality in Taiwan. Acta Diabetol 50: 879–886. 23508375 10.1007/s00592-013-0471-0

[18] Tseng CH (2013) Type 2 diabetes, smoking, insulin use and mortality from hepatocellular carcinoma: a 12-year follow-up of a national cohort in Taiwan. Hepatol Int 7: 693–702. 26201803 10.1007/s12072-012-9405-0

[19] Tseng CH (2013) Insulin use and smoking jointly increase the risk of bladder cancer mortality in patients with type 2 diabetes. Clin Genitourin Cancer 11: 508–514. 23791436 10.1016/j.clgc.2013.04.019

[20] Tseng CH (2012) Pioglitazone and bladder cancer: a population-based study of Taiwanese. Diabetes Care 35: 278–280. 22210574 10.2337/dc11-1449 PMC3263866

[21] Tseng CH, Tseng FH (2012) Peroxisome proliferator-activated receptor agonists and bladder cancer: lessons from animal studies. J Environ Sci Health C Environ Carcinog Ecotoxicol Rev 30: 368–402. 23167631 10.1080/10590501.2012.735519

[22] Tseng CH (2014) A review on thiazolidinediones and bladder cancer in human studies. J Environ Sci Health C Environ Carcinog Ecotoxicol Rev 32: 1–45. 24598039 10.1080/10590501.2014.877645

[23] Tseng CH (2013) Rosiglitazone is not associated with an increased risk of bladder cancer. Cancer Epidemiol 37: 385–389. 23619142 10.1016/j.canep.2013.03.013

[24] Klubo-Gwiezdzinska J, Jensen K, Costello J, Patel A, Hoperia V, et al (2012) Metformin inhibits growth and decreases resistance to anoikis in medullary thyroid cancer cells. Endocr Relat Cancer 19: 447–456. 22389381 10.1530/ERC-12-0046

[25] Chen G, Xu S, Renko K, Derwahl M (2012) Metformin inhibits growth of thyroid carcinoma cells, suppresses self-renewal of derived cancer stem cells, and potentiates the effect of chemotherapeutic agents. J Clin Endocrinol Metab 97: E510–520. 22278418 10.1210/jc.2011-1754

[26] Vigersky RA, Filmore-Nassar A, Glass AR (2006) Thyrotropin suppression by metformin. J Clin Endocrinol Metab 91: 225–227. 16219720 10.1210/jc.2005-1210

[27] Díez JJ, Iglesias P (2013) Relationship between serum thyrotropin concentrations and metformin therapy in euthyroid patients with type 2 diabetes. Clin Endocrinol (Oxf) 78: 505–511. 22686474 10.1111/j.1365-2265.2012.04468.x

[28] Rezzónico J, Rezzónico M, Pusiol E, Pitoia F, Niepomniszcze H (2011) Metformin treatment for small benign thyroid nodules in patients with insulin resistance. Metab Syndr Relat Disord 9: 69–75. 21128816 10.1089/met.2010.0026

[29] Rizos CV, Elisaf MS (2013) Metformin and cancer. Eur J Pharmacol 705: 96–108. 23499688 10.1016/j.ejphar.2013.02.038

[30] Tseng CH (2014) Treatment with human insulin does not increase thyroid cancer risk in patients with type 2 diabetes. Eur J Clin Invest 44: 736–742. 24931333 10.1111/eci.12290

[31] Kourelis TV, Siegel RD (2012) Metformin and cancer: new applications for an old drug. Med Oncol 29: 1314–1327. 21301998 10.1007/s12032-011-9846-7

[32] Margel D, Urbach DR, Lipscombe LL, Bell CM, Kulkarni G, et al (2013) Metformin use and all-cause and prostate cancer-specific mortality among men with diabetes. J Clin Oncol 31: 3069–3075. 23918942 10.1200/JCO.2012.46.7043

[33] Willey CJ, Andrade SE, Cohen J, Fuller JC, Gurwitz JH (2006) Polypharmacy with oral antidiabetic agents: an indicator of poor glycemic control. Am J Manag Care 12: 435–440. 16886886

[34] Aljada A, Mousa SA (2012) Metformin and neoplasia: Implications and indications. Pharmacol Ther 133: 108–115. 21924289 10.1016/j.pharmthera.2011.09.004

[35] Ittermann T, Markus MR, Schipf S, Derwahl M, Meisinger C, et al (2013) Metformin inhibits goitrogenous effects of type 2 diabetes. Eur J Endocrinol 169: 9–15. 23572084 10.1530/EJE-13-0101

[36] Cappelli C, Rotondi M, Pirola I, Agosti B, Formenti A, et al Thyreotropin levels in diabetic patients on metformin treatment. Eur J Endocrinol 167: 261–265. 22645202 10.1530/EJE-12-0225

[37] Nar A, Gedik O (2009) The effect of metformin on leptin in obese patients with type 2 diabetes mellitus and nonalcoholic fatty liver disease. Acta Diabetol 46: 113–118. 18839053 10.1007/s00592-008-0067-2

[38] Klubo-Gwiezdzinska J, Costello J Jr, Patel A, Bauer A, Jensen K, et al (2013) Treatment with metformin is associated with higher remission rate in diabetic patients with thyroid cancer. J Clin Endocrinol Metab 98: 3269–3279. 23709654 10.1210/jc.2012-3799

[39] Malaguarnera R, Frasca F, Garozzo A, Gianì F, Pandini G, et al (2011) Insulin receptor isoforms and insulin-like growth factor receptor in human follicular cell precursors from papillary thyroid cancer and normal thyroid. J Clin Endocrinol Metab 96: 766–774. 21123448 10.1210/jc.2010-1255

[40] Würth R, Pattarozzi A, Gatti M, Bajetto A, Corsaro A, et al (2013) Metformin selectively affects human glioblastoma tumor-initiating cell viability: A role for metformin-induced inhibition of Akt. Cell Cycle 12: 145–156. 23255107 10.4161/cc.23050 PMC3570504

[41] Würth R, Barbieri F, Florio T (2014) New molecules and old drugs as emerging approaches to selectively target human glioblastoma cancer stem cells. Biomed Res Int 2014: 126586. 24527434 10.1155/2014/126586 PMC3909978

[42] Tseng CH (2013) Diabetes and thyroid cancer mortality: a 12-year prospective follow-up of Taiwanese. Eur J Clin Invest 43: 595–601. 23550697 10.1111/eci.12086

[43] Shih SR, Chiu WY, Chang TC, Tseng CH (2012) Diabetes and thyroid cancer risk: literature review. Exp Diabetes Res 2012: 578285. 22778714 10.1155/2012/578285 PMC3384966

